# Effects of E-Cigs on Physiological Pathways and Proposed Therapeutic Intervention with Bixin

**DOI:** 10.3390/biomedicines12122705

**Published:** 2024-11-27

**Authors:** Sophia Rene Claymore, Diane S. Allen-Gipson

**Affiliations:** 1 Department of Pharmaceutical Sciences, USF Health Taneja College of Pharmacy, University of South Florida, Tampa, FL 33612, USA; srclaymore@usf.edu; 2 Department of Internal Medicine, Division of Pulmonary, Critical Care, & Sleep, University of Nebraska Medical Center, Omaha, NE 68198, USA

**Keywords:** e-cigs, bixin, nanoparticles, inflammation

## Abstract

Electronic cigarettes (e-cigs) have increased in popularity and usage over the last few decades. There is rising concern regarding the long-term effects of e-cigs on human health, considering their relatively recent introduction to the market. E-cigs are generally composed of a liquid containing nicotine and various chemicals, a battery, a vaporization chamber, and a coil that serves to heat the liquid upon inhalation of the mouthpiece. While e-cigs were initially introduced as a healthy alternative to cigarette smoking, recent research has demonstrated the cytotoxic effects of nicotinic e-cig devices on multiple cell types, including epithelial and endothelial cells, along with causing dysregulation of inflammatory pathways. This review will discuss the harmful effects of e-cigs on the human body, highlighting the physiological impact of e-cigs on pulmonary, cardiovascular, and cerebrovascular health. Moreover, this review will highlight the potential therapeutic effects of bixin, an apocarotenoid found in the seeds of *Bixa orellana,* also known as the achiote tree, due to its innate anti-inflammatory, antioxidant, and anti-cancer activities that have been demonstrated in recent research. Nanotechnology has surfaced in the past few decades as a powerful tool for medicinal practice. Specifically, nanoparticles serve as a potential method for treating various conditions and diseases. Bixin nanoparticles show promise as a viable method for treating e-cig-induced damage due to the innate properties of bixin and the advantages of using nanoparticles compared to conventional medicinal interventions.

## 1. Introduction: Electronic Cigarettes vs. Conventional Cigarettes

Electronic cigarettes (e-cigs) were first introduced to the market in the early 2000s as a “healthy” alternative to cigarettes and as a viable method to quit smoking conventional cigarettes. Consequently, companies in the United States and the United Kingdom altered their marketing strategies between 2014 and 2017, leading to an increase in sales of e-cigs by 146% [1]. As e-cigs became more popular, companies attempted to increase the addictive nature of these devices by increasing the nicotine content. As a result, the JUUL pod was introduced, resulting in a 640% increase in e-cig sales [1]. These nicotinic devices are now available in a wide variety of sizes with differing nicotine concentrations and flavors, allowing e-cigs to cater to a broad audience.

Despite the introduction of e-cigs as a “healthy” alternative to conventional cigarette users, research has shown that “some e-cig users were able to attain tobacco cigarette-like peak plasma nicotine levels” [2]. This notion suggests that similar amounts of nicotine are delivered systemically by both e-cigs and conventional cigarettes. However, similar nicotinic delivery may be an incentive for conventional tobacco cigarette users to quit smoking with the knowledge that “over 4000 harmful and potentially harmful constituents (HPHCs) have been identified in traditional cigarette smoke” [3]. In contrast, the HPHCs in e-cig aerosol are between 9 and 450 times lower than within conventional tobacco cigarettes [3].

Although the harmful constituents found in conventional tobacco cigarettes are at lower levels in e-cigs, the pharmacokinetic parameters of e-cigs are very similar to those of tobacco cigarettes [2]. An important parameter is the maximum drug concentration in plasma (C_max_), representing the amount/concentration of drug observed in plasma or blood samples [4]. Previous research shows that the average C_max_ of nicotine after e-cig use was 8.4 ng/mL, which is lower than the average C_max_ range in tobacco cigarette users (approximately 11 ng/mL); however, some users in the study achieved C_max_ levels within the typical range of tobacco cigarette smokers [2]. The systemic retention of nicotine from e-cigs was found to be higher (94%) than the average retention of nicotine from tobacco cigarettes (which ranges between 80 and 90%). Additionally, a significant amount of nicotine from e-cigs is absorbed in sites other than the lungs, such as the “buccal mucosa and gastrointestinal tract following swallowing” [2]. This systemic absorption explains the lower C_max_ nicotine levels of e-cig users compared to tobacco cigarette users.

The systemic absorption of nicotine from e-cigs into areas other than the lungs presents the possibility that metal particles, silica particles, and harmful aldehydes are also absorbed into different tissues, causing systemic inflammation and ROS generation. For example, research confirmed the presence of toxic chemicals such as iron, manganese, zinc, and copper in both the e-liquid and aerosolized e-liquid; iron and copper can cause respiratory irritation, while manganese and zinc can cause reduced lung function [5]. These researchers suggest that metals are transferred from the e-cig coil to the e-liquid and subsequently from the e-liquid to the aerosolized vapor following the heating of the coil upon inhalation [5]. Collectively, these research findings stress the dangers of e-cigs compared to conventional tobacco cigarettes, regardless of the lower average e-cig C_max_ nicotine values following inhalation.

## 2. Nicotinic Damage on the Lungs

Nicotine is the major component of e-cig and cigarette smoke (CS); it is metabolized into other constituents, which further affect the body. Most (80%) of the inhaled nicotine is metabolized to cotinine, excreted into urine, and considered non-toxic and non-carcinogenic [6]. However, some inhaled nicotine is metabolized into nicotine-derived nitrosamine ketone (NNK) and nitrosonornicotine (NNN). NNK is further metabolized into methyldiazohydroxide (MDOH), a pyridyl-butyl derivative (PBD), and formaldehyde, while NNN is degraded into hydroxyl or keto PBDs [6]. These metabolites can interact with DNA and other molecules, further causing damage. For example, researchers observed that mice exposed to 12 weeks of e-cig vapor showcased DNA damage in both human lung epithelial and bladder urothelial cells [7]. Furthermore, these metabolites are highly carcinogenic compounds within the systemic circulation, causing “mutations to critical human DNA genes” [8]. Moreover, the levels of two crucial proteins for nucleotide-excision repair (NER) and base-excision repair (BER) have been found to significantly decrease in the lung tissues of mice exposed to CS [6].

Research has found that the small size of e-cig aerosol allows for deep penetration into lung tissues, causing DNA damage in bronchoalveolar cells [7]. This DNA damage can be quantified by measuring levels of nicotine metabolites in different organs. One study found mutagenic guanosines in mice’s lungs, bladder, and heart after e-cig exposure [6]. These results showcase that the metabolization of nicotine into harmful nitrosamines occurs in vivo in mice. Additionally, researchers found that nicotine and its metabolite NNK impact human bronchial epithelial and urothelial cells by enhancing their “mutational susceptibility and tumorigenic transformation” [6]. The damage found in mice following e-cig exposure, combined with the impact of nicotine metabolites on cultured epithelial cells, potentiates the possibility of tumorigenesis within in vivo humans exposed to e-cigs. 

The introduction of nicotine to the human body results in a cascade of harmful effects, regardless of whether the nicotine is inhaled through an e-cig or a conventional tobacco cigarette. Recent research on e-cigs has demonstrated that both the e-liquid and the aerosolized e-vapor cause damage to lung tissue and cells, regardless of the presence of nicotine. This research investigated the effects of e-cig vapor extract and nicotine-free e-cig vapor extract (NF ECV) on epithelial cells and pulmonary arterial smooth muscle cells [9]. Significant changes occur in lung structure and function after e-cig usage, independent of the presence of nicotine.

While the inflammatory effects and structural alterations occurred to a lesser degree with the introduction of nicotine-free e-cig vapor extract (NF ECV), the components of NF ECV still induce “cell type-specific effects and mild pulmonary alterations” [9]. The inhalation of NF ECV and ECV increases endothelial permeability and induces inflammatory responses in the lungs; this inflammatory response includes increased levels of neutrophils and lymphocytes following both NF ECV and ECV exposure [9]. A longitudinal cohort pilot study also found that “circulating monocytes from e-cig users had alterations in their inflammatory phenotype” even with reduced e-cig usage [10]. These studies suggest that e-cig users have a decreased ability to respond to infection even after a decrease in e-cig usage.

Additionally, research revealed that when a normal epithelial cell line (HaCaT) and head and neck squamous cell carcinoma cell (HNSCC) lines are exposed to e-cig vapor in the absence of nicotine, the e-cig aerosol induced a 5-fold increase in cell death [11]. Comparatively, exposure to e-cig vapor containing nicotine induced a 10-fold increase in cell death in these cell lines. Furthermore, significant DNA strand breaks were observed even without nicotine [11]. E-cig vapor extract induces “significant endothelial damage, inflammation, and parenchymal alterations,” holistically demonstrating that the inclusion of nicotine magnifies the damaging components of nicotine-free e-cigs [9].

In turn, in a similar experiment, in which mice were exposed to e-cig aerosol and CS, the researchers found that the “peripheral vasoconstriction response was similar between mice exposed to e-cig aerosol or e-cig [vapor]” in a nicotine-independent response, regardless of whether the base solution of the e-cig [12]. These research studies showcase that even without nicotine, e-cigs still cause damage to the respiratory tract and induce functional alterations in important anti-inflammatory molecules. Furthermore, these studies demonstrate components of nicotine-free e-cigs can cause harmful physiological responses to the vasculature and immunomodulatory molecules.

## 3. E-Cig Flavoring Chemicals

Recent researchers have highlighted the relationship between e-cig flavorings and harmful chemicals associated with these different flavors [12,13,14]. Typically, e-cigs contain a base solution consisting of propylene glycol, vegetable glycerin, and nicotine [12]. Several chemicals can then be added to propylene glycol, vegetable glycerin, and nicotine mixtures to introduce flavoring to the e-liquid [12]. The different flavoring of e-cigs has served to attract younger users, especially if these flavors are sweet without the harsh smell associated with traditional cigarettes [15]. Researchers have investigated the change in conventional cigarette usage as a function of e-cig flavor [13,15]. They found that chocolate-flavored e-cigs were associated with the lowest drop in e-cig usage amongst traditional cigarette smokers [13]. In contrast, menthol-flavored e-cigs contributed to the most significant drop in traditional cigarette usage [13]. Conversely, tobacco- and cherry-flavored e-cigs resulted in the highest levels of e-cig usage [13].

Traditional smokers typically have an increased usage of menthol-flavored e-cigs due to the nature of menthol and its action on nicotinic acetylcholine (nACh) receptors [16]. Menthol modulates the function of α7-nACh receptors by potentially acting as a non-competitive antagonist, suggesting that menthol flavorings are more attractive to both traditional cigarette and e-cig users due to their ability to trigger the cold-sensitive transient receptor potential melastatin (TRPM) receptor [16]. This action is the primary mechanism by which menthol provides a cooling sensation when inhaled or applied to the skin. Upon chronic or acute administration of menthol, the standard nicotine-induced decreases in body temperature are diminished [16]. While the usage of menthol-flavored e-cigs may contribute to a reduction in traditional cigarette usage, the composition of e-liquids that result in different flavors is full of harmful chemicals.

The flavoring chemicals within e-cigs are biologically active compounds called aldehydes, where different chemicals can cause differential damage to human macrophages and bronchial epithelial cells [14]. For example, 2,3-butanedione, a commonly used chemical to provide a buttery flavoring, “was found to cause severe inflammation and scarring of the bronchioles when inhaled” [14]. At the same time, ethyl maltol (sweet, sugary) was “found to generate free radicals when vaporized” [14]. These researchers investigated the cytotoxic and inflammatory effects of thirty different e-cig flavoring chemicals, showcasing the generation of reactive oxygen species (ROS), heightened inflammation, and the cytotoxicity of these chemicals. Their findings stressed the importance of understanding how the aerosolization of these chemicals can differentially affect epithelial cells and macrophages in vivo on a long-term scale, especially considering that this study was conducted with the chemicals in liquid form.

**Figure 1:**
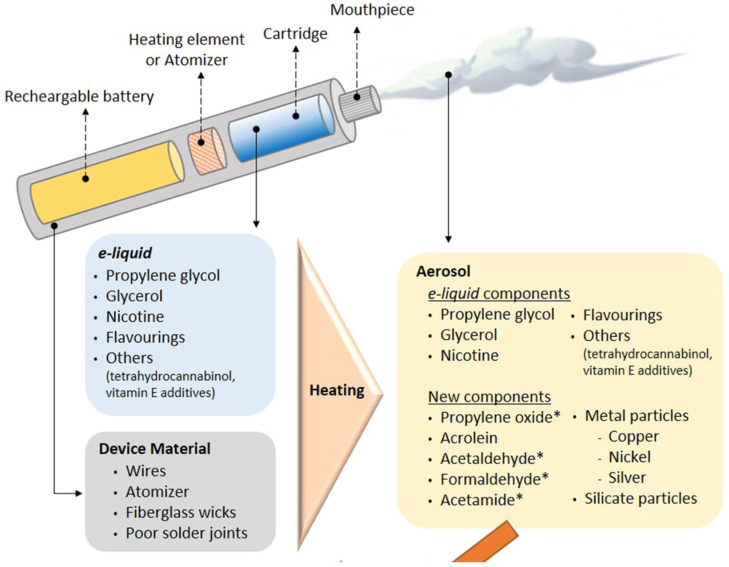
Graphic image demonstrating the components found in e-cig devices, including the e-liquid and the device material. This image also showcases how the heating of the e-liquid and device introduces aerosolized components of the e-liquid and other new components into the aerosol, which is inhaled by the e-cig user [17]. The * defines human carcinogens or potent human carcinogens.

While studies have determined the effects of e-cig flavoring chemicals in liquid form, other researchers have showcased how the aerosolization of e-liquids generates new components. Heating the e-liquid and the device itself can lead to inhaling acetaldehyde, formaldehyde, metal particles, and silicate particles not detected within the cooled e-liquid as depicted in Figure 1 [17].

In turn, the “levels of aldehydes and methyl ethyl ketone [are] significantly higher (2–125) times in exhaled e-cig breaths” compared to before e-cig inhalation [18]. E-cig breath refers to the exhale of a user following inhalation from an e-cig device; these researchers collected the human breath of participants following e-cig inhalation and without e-cig inhalation to measure the differences in aldehyde presence between e-cig and non-e-cig users. They also determined that the “mean retention of formaldehyde in the respiratory tract was 99.7% for all participants, while acetaldehyde retention was 91.6%” [18]. These generated components from e-cig inhalation are of most significant concern, as they cause increased respiratory complications and cardiovascular risk due to the generation of ROS, the stiffening of arterioles, and impairment of both epithelial and endothelial function [17]. Due to the hydrophilic properties of aldehydes and formaldehyde, they are rapidly taken up by the respiratory tract because of the hydrophilic surface of lung tissues [18]. This rapid uptake of aldehydes and formaldehyde induces a pulmonary inflammation response.

The aerosolization of e-cig liquid introduces various metal particles to lung tissue, such as copper, nickel, and silver, along with silicate particles contributing to lung injuries [17]. Inhalation of silica particles causes “prolonged cycles of massive inflammation, epithelial hyperplasia, and the formation of dense nodules with a central hyalinized whorled collagen in the lungs” [19]. Furthermore, within the lungs, alveolar macrophages are responsible for eliminating harmful substances from epithelial tissue along the respiratory tract; however, when the silica particles are taken up and phagocytosed by macrophages, there is an increased generation of reactive oxygen species (ROS), which further contribute to increased inflammation in the lungs [19]. Even without the knowledge of how usage of these devices can affect health over a more extended period, the current understanding of using e-cig suggests dangerous consequences for e-cig users.

## 4. Effects of E-Cigs on Adolescents and Young Adults

Since the introduction of e-cigs as an alternative to cigarettes, e-cigs have been generally consumed by younger populations. With the concurrent rise and ease of social media, e-cig consumption by younger populations has hurt the health of adolescents. A logistic regression model analysis was conducted on adolescent vaping status by researching teenage usage of social media platforms (Instagram, Snapchat, Twitter, and Facebook) [20]. The model analysis demonstrated that the usage of these platforms was significantly associated with vaping status [20]. The usage of e-cigs by younger populations is a significant concern because of the lack of knowledge regarding the long-term effects of e-cig usage on developmental health.

During development, human brains undergo synaptic pruning, in which neuronal connections change over time, eliminating unnecessary connections and building new ones. The brain is not fully developed until approximately twenty-five years of age. However, many factors, such as the environment, epigenetics, and drug usage, can affect this process, highlighting the colloquial phrase “nature vs. nurture” [3].

Nicotine, a highly addictive chemical, and its introduction and usage of nicotine by adolescents may disrupt the process of synaptic pruning, potentially disrupting the proper development of crucial neuronal circuitry, such as the reward system [3]. Studies have shown that adolescents may demonstrate greater sensitivity to the effects of nicotine (along with other addictive chemicals) because their neuronal circuitry is not fully matured [3]. This developmental disruption can be further magnified by the usage of alcohol along with nicotinic e-cigs [21].

One study investigated the perceived pleasure reported by young adults when using e-cigs and drinking alcohol; the young adults in this study reported that they felt increased pleasure from using e-cigs in combination with alcohol usage [21]. Interestingly, they reported a greater sense of pleasure from using cigarettes compared to e-cigs with alcohol consumption [21]. Furthermore, this increase in rewarding effects may also increase the likelihood of abusing both substances.

The potential abuse of e-cigs is already high due to the addictive nature of nicotine. Yet, the combined usage of e-cigs and alcohol suggests the risk of developing long-term substance abuse problems. The relationship between e-cig usage and alcohol consumption could potentiate that “e-cig users are more likely to participate in hazardous drinking and are at higher risk for alcohol use disorder” [22]. The results from these studies highlight the importance of implementing health interventions regarding the harmful effects and abuse liability of e-cigs amongst younger populations.

Health interventions primarily involve informational sessions for students and younger populations, but they can also include parent education on the harmful effects of e-cig usage on adolescent brain development. In the early 1990s, similar informational sessions were implemented in primary educational settings with the campaign “Just Say No” to discourage adolescents from participating in drug use [23]. The lack of success associated with these campaigns is attributed to the lack of education regarding how these drugs negatively affect young minds [23]. Additionally, these campaigns did not provide information on how young people can safely use drugs if they decide to do so. To learn from the mistakes of these unsuccessful campaigns, it is essential to implement educational resources on why and how e-cig usage is harmful and dangerous to stress non-usage in younger populations.

In addition to the general harmful effects of nicotinic e-cig usage on adolescents, research has also shown that there are neuronal sexual differences in the withdrawal effects of e-cig users [24]. The sexual differentiation of withdrawal from nicotine can be studied by focusing on Fos expression within the interpeduncular nucleus (IPN) [24]. The IPN is “a midbrain structure situated in the ventral portion of the tegmentum, where the ventral portion modulates negative affective states, whereas the rostral portion modulates physical signs produced by nicotine withdrawal” [24]. The IPN has been linked to addiction and anxiety-like behaviors through addiction and withdrawal research of substances such as methamphetamines and nicotine [25].

This portion of the brain is a valuable source of information that can be used to deduce the degree to which withdrawal occurs. The expression of Fos is commonly used in neurobiological research due to the correlation of activated neurons and increased Fos expression in response to specific stimuli [26]. In the IPN, increased Fos expression or the presence of Fos-positive cells indicates higher levels of IPN activity [24]. One research study found that physical withdrawal symptoms in mice resulting from mecamylamine-precipitated withdrawal are associated with increased levels of Fos expression in the IPN [27]. In turn, this increased Fos expression is associated with higher levels of GABAergic neuron activity in the IPN, where GABAergic neurons in the IPN are involved with regulating drug withdrawal symptoms and anxiety-like behaviors [27].

Research has shown that while both male and female mice display observable Fos expression in the IPN, female mice exhibited higher levels of Fos-positive cells [24]. This research suggests that there is a portion of the IPN that “plays a role in modulating sex differences in negative affective states produced by withdrawal” [24]. Even though it has been demonstrated that nicotinic e-cigs are addictive and induce withdrawal-like behaviors in both male and female mice, research suggests that female e-cig users may face a more significant challenge in the cessation of e-cig usage [24]. Female e-cig users may struggle more with e-cig usage cessation due to the increased levels of Fos expression and its consequential association with increased GABAergic neuron activity. This heightened GABAergic neuronal activity is linked to more significant drug withdrawal symptoms and anxiety-like behaviors.

There have already been reports of extreme damage in young adults caused by high e-cig usage [28]. A case study demonstrated the incredibly harmful effects of nicotine by describing the rapid and fatal progression of human papillomavirus (HPV)-negative squamous cell carcinoma in a young adult [29]. The study revealed a patient with an oral cavity typically seen in older patients with long histories of alcohol and tobacco use, as compared to the typical presentation of HPV-positive patients seen more commonly in younger populations [29]. Although this patient did have an extensive history of cigarette and e-cig usage, the potential for developing such a condition remains prevalent, as current e-cig users are generally unaware of the long-term effects of e-cig usage [28].

Nicotinic e-cigs cause pro-oncogenic activity due to the activation of epidermal growth factor (EGF) activation [30]. Researchers investigated the modulation of fatty acid synthase (FASN) activity in response to nicotine [30]. FASN is an enzyme that controls the final step of de novo lipogenesis, contributes to “membrane biosynthesis, energy storage, and lipid signaling necessary for malignant cell growth”, and may also activate epidermal growth factor receptors (EGFR), which is a joint event in oral carcinogenic patients [30]. They observed that introducing nicotine to oral dysplastic cells caused a significant upregulation of FASN, leading to the activation of EGFR and increased cell migration [30]. Collectively, this research further suggests that e-cig usage contributes to pro-oncogenic activities, even in young adults, specifically regarding oral squamous cell carcinoma [29,30].

## 5. E-Cig-Mediated Oxidative Stress: NRF2 and iNOS Pathways

To properly understand the harmful effects of e-cig-induced oxidative stress on the physiological function of cardiovascular, pulmonary, and cerebrovascular systems, it is necessary to provide an overview of the nuclear factor erythroid 2-related factor 2 (NRF2) and nitric oxide synthase (NOS) pathways.

Multiple signaling pathways in the human body are designed to protect against the introduction of oxidative stress. Specifically, the Kelch-like ECH-associated protein 1 (KEAP)-NRF2 pathway serves as the “principal protective response to oxidative and electrophilic stresses” [31]. NRF2 also serves as “one of the most functionally important transcription factors related to the endogenous antioxidant defense system” due to its “crucial role in the neutralization of ROS [32]. The NRF2 pathway achieves neutralization of ROS through the regulation of anti-oxidative genes such as NAD(P)H quinone oxidoreductase 1 (NQO1) and heme-oxygenase-1 (HO-1) [33]. This pathway can respond to different stimuli to activate its cytoprotective activity: “(1) chemical inducers of NRF2 activity, (2) KEAP1, the protein sensor of these components, (3) the transcription factor NRF2, which modulates the transcriptional response to inducers and oxidative stress, and (4) the target genes, which provide the cytoprotective output of the pathway” [31].

Chemical inducers of NRF2 activity can include dietary compounds, pharmaceuticals, oncometabolites, and endogenous signaling compounds such as hydrogen peroxide and nitric oxide [31]. KEAP1 was found to be the protein sensor of chemical inducers through experimentation involving the consequences of its overexpression and downregulation: the overexpression of KEAP1 represses the activity of NRF2, while chemical inducers reverse this repression [31]. NRF2 was determined to be the primary factor in the antioxidant response through various experiments; one experiment showed that the induction of an antioxidant stress response was eliminated without NRF2 [31].

Additionally, the activity of NRF2 directly correlates with the expression of target genes, allowing for antioxidant and cytoprotective activities as a function of the immune response [31]. Furthermore, the expression of antioxidant-generating enzymes and xenobiotic-metabolizing enzymes caused by NRF2 expression allows the antioxidative reaction to occur [31]. Under homeostatic conditions, NRF2 is at a low level “due to its KEAP1-mediated proteosome-dependent degradation” [31]. NRF2 is bound to KEAP1, maintaining what is known as the KEAP1-NRF2 complex, and glutathione functions as an ever-present scavenger of naturally produced ROS. In these basal conditions, the E3 ubiquitin ligase is a component of KEAP1 that regulates NRF2 activity by “targeting it for ubiquitination and proteasome-dependent degradation” [31].

However, under oxidative or electrophilic stress, NRF2 can dissociate from KEAP1 because sensor cysteines in KEAP1 allow NRF2 to escape ubiquitination [31]. Thus, degradation of NRF2 is inhibited without the activity of the E3 ubiquitin ligase. Consequently, “NRF2 dissociates from KEAP1, accumulates in the cell, translocates into the nucleus, and binds to the antioxidant response element (ARE), promoting the transcription of the target gene” [34]. The target gene, after transcription, will introduce antioxidant and detoxifying proteins and molecules that will lessen the oxidative stress presented to the system.

The NOS pathway can be divided into different components based on its differential mechanisms of action in response to various stimuli, including e-cigs. The body produces endothelial NOS (eNOS), neuronal NOS (nNOS), and inducible NOS (iNOS). The main commonality between these pathways is that “in the presence of nicotinamide adenine dinucleotide phosphate (NADPH), a proton, tetrahydrobiopterin (H4B), and dioxygen, NOS catalyzes the generation of nitric oxide (NO) from the natural substrate L-arginine, where NO and L-citrulline are produced after arginine oxidation” [35].

Nitric oxide is a crucial vasoprotective cellular signaling molecule that regulates vascular tone [36]. NO is an important cellular signaling molecule at low concentrations; the dysregulation of NO production can result in oxidative stress because “the production of adequate levels of NO in the vascular endothelium is critical for the regulation of blood flow and vasodilation” [37].

Neuronal NOS plays a role in “synaptic plasticity in the central nervous system, central regulation of blood pressure, smooth muscle relaxation, and vasodilation via peripheral nitrergic nerves” [38]. Endothelial NOS helps maintain the homeostasis of endothelial cells as it “keeps blood vessels dilated, controls blood pressure, and has numerous other vasoprotective and anti-atherosclerotic effects” [38]. In basal conditions, eNOS is the primary contributor of NO to the body [37]. Finally, the inducible NOS is typically activated because of a harmful stimulus, as it “contributes to the pathophysiology of inflammatory diseases” and “can be expressed in many cell types in response to lipopolysaccharide, cytokines, or other agents” such as ROS [38]. The nuclear factor-kappa B (NF-κB) pathway acts upstream of the iNOS pathway and “plays a crucial role in the inflammatory response and production of pro-inflammatory molecules”, including iNOS [39].

The eNOS and iNOS pathways are essential to regulating the generation of ROS throughout the body, especially the lungs. While these NOS pathways are active throughout the body, for this literature review, the focus will be predominantly on the activities of eNOS and iNOS in the lungs in response to nicotine- and nicotine-free e-cigs along with traditional cigarettes.

## 6. Effects of E-Cigs on the NRF2 and iNOS Pathways

Cigarette smoke enhances iNOS expression through activation of the NF-κB pathway, while cigarette smoke extract (CSE) induces inhibition of the NRF2/HO-1 pathway and activation of the NF-κB pathways [33,36]. Comparable effects on both the NRF2 pathway and the NF-κB pathway occur after exposure to e-cig vapor.

**Figure 2:**
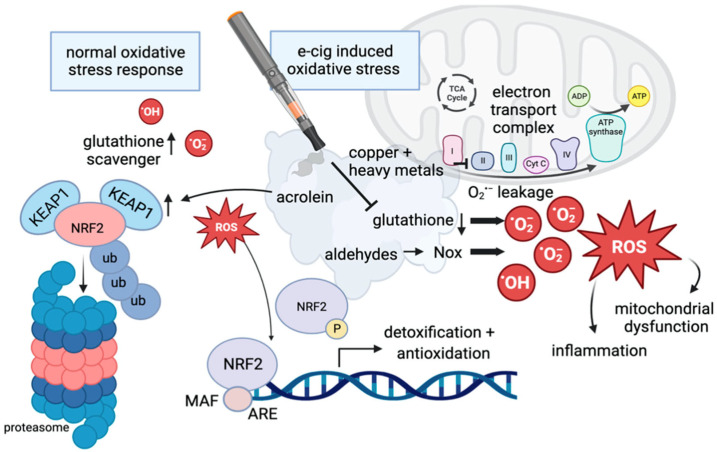
Graphic demonstrating the effects of e-cigs on inflammatory pathways. Under basal conditions, KEAP1 is bound to NRF2, where NRF2 is a target of ubiquitination and proteasome-dependent degradation. Additionally, glutathione functions as a scavenger of free radicals. Under e-cig-induced conditions of oxidative stress, NRF2 dissociates from KEAP1 and enters the nucleus to initiate transcription of antioxidant gene expression (ARE). The introduction of aldehydes, copper, and heavy metals interferes with glutathione function, decreasing its activity. The dysregulation of glutathione increases the presence of ROS, leading to mitochondrial dysfunction and inflammation [3].

Under oxidative stress following exposure to e-cig vapor, glutathione levels are diminished, and NRF2 separates from KEAP1. It binds to ARE to promote antioxidant transcription; consequently, iNOS expression is enhanced, and eNOS expression is repressed. While the separation of NRF2 from KEAP1 and subsequent antioxidant transcription indicates the activation of the KEAP1-NRF2 pathway, following e-liquid aerosolization and inhalation, NRF2 antioxidative function and KEAP1 expression are disrupted as depicted in Figure 2 [3]. Similarly, cigarette extract exposure suppresses the protein expression of NRF2 and further downstream expression of the HO-1 gene [33].

The NRF2 and iNOS pathways are designed to reduce levels of oxidative stress, but the inhalation of e-cig vapor (1) decreases the antioxidative regulatory mechanisms of the KEAP1-NRF2 pathway and (2) causes overproduction of NO through the upregulated expression of iNOS [40]. The iNOS pathway can generate higher levels of NO compared to both eNOS and nNOS [37]. The overproduction of NO by iNOS enzymes is supposed to serve as an immune response to restore basal conditions. However, the continuous upregulation of iNOS as an inflammatory response to e-cig-induced damage leads to impairment of vascular function because the excess amounts of circulating NO decrease NO sensitivity [37].

Without the activation of the KEAP1-NRF2 pathway, ROS accumulate within the human body. Prolonged periods of oxidative stress can damage DNA and lead to many diseases, including cancer, atherosclerosis, and neurodegeneration [40]. The downregulation of HO-1 expression increases ROS generation, increasing oxidative stress and consequential inflammatory damages [41].

Furthermore, the concurrent repression of eNOS and enhanced expression of iNOS causes imbalances. This dysregulation of eNOS expression has been linked to “disorders including oxidative stress, endothelial dysfunction, [and] vascular diseases,” especially considering that eNOS plays a pivotal role in the regulation of cardiovascular anti-proliferative and anti-apoptotic actions [35]. Reduced eNOS expression mediated by CS exposure revealed “endothelial pyroptosis and impaired vascular relaxation” [36]. Pyroptosis is a cell-mediated necrosis and is a function of both enhanced iNOS expression and inhibited eNOS expression [36]. It has been found that CS exposure-induced pyroptosis occurs in endothelial cells as a response to impaired aorta relaxation and vascular dysfunction [36]. The simultaneous reduction in eNOS expression and enhancement of iNOS expression, along with increased levels of oxidative stress resulting from CS exposure, induces pyroptosis in endothelial cells and increases vascular damage.

Research has been conducted to focus on how CS impairs cardiovascular function, along with investigations of the differential endothelial responses from e-cig and conventional cigarette users using non-users as a control [36,42]. Findings revealed that e-cig usage, but not cigarette usage, “causes changes in the blood that increase microvascular endothelial permeability and may have a vaping-specific effect on intracellular oxidative state” [42]. Additionally, cells treated with sera from e-cig users contained less eNOS protein than cells treated with sera from conventional cigarette smokers [42]. At the same time, the enhanced activation of iNOS suggests higher production of NO to facilitate the increase in ROS, in which “overproduction of NO by iNOS has been implicated in inflammation, rheumatoid arthritis, infection susceptibilities, irritable bowel syndrome (IBS), immune-type diabetes, stroke, sepsis, thrombosis, cancer, and multiple sclerosis (MS)” [35].

Research regarding the damaging effects of e-cig aerosol on vascular function and endothelial cells showcases many similarities between that of cigarette smoke exposure [42]. The NRF2 and iNOS pathways have been consistently indicated as essential mediators of oxidative stress and vascular function, and the research mentioned has shown that e-cig aerosol exposure dysregulates both pathways along with eNOS production. The long-term effect of this dysregulation is unknown. Still, the short-term consequences of e-cig aerosol exposure demonstrate heightened ROS levels, decreases in antioxidative mechanisms, and increases in vascular, epithelial, and endothelial damage [35,36,40,42].

More research is needed to understand the underlying mechanisms by which the NRF2 pathway and simultaneous iNOS enhancement and eNOS repression occur following e-cigarette exposure. Most current studies describe the effects of cigarette smoke or cigarette smoke extract on these pathways. Still, more information is needed to clarify how conventional cigarettes affect these pathways in tandem. A greater understanding of these underlying mechanisms will increase the likelihood of successfully utilizing therapeutic intervention to lessen the damages that occur following e-cigarette exposure, regardless of the presence of nicotine.

## 7. Physiological Effects of E-Cigs on Systemic Function

### 7.1. Cytokines

It is essential to understand the physiological effects of how e-cigs alter the immune functions of pulmonary, cerebrovascular, and cardiovascular systems and their ability to induce DNA damage. A longitudinal study explored the changes in the inflammatory state and monocyte function following e-cig exposure compared to non-user controls [10]. The researchers quantified the presence of 38 different proteins. They found that “airway samples from e-cig users tended to have decreased levels of immunomodulatory proteins relative to healthy controls, whereas levels of cytokines, chemokines, and growth factors in circulation tended to be elevated” [10]. Inflammatory cytokines include molecules like tumor necrosis factor alpha (TNF-α), interleukin (IL)-6, IL-8, and IL-1, in which increased levels of both cytokines and chemokines indicate a physiological attempt to decrease an inflammatory stimulus. A greater understanding of the role of these cytokines and chemokines and their inflammatory response caused my nicotinic e-cig-induced damage. Recent research has demonstrated dysregulation of cytokine and chemokine activity following e-cig exposure, notably including downregulation of important receptors [43,44].

The IL-22 receptor (IL-22R-α1) is downregulated in the presence of nicotine [43]. IL-22 helps maintain lung homeostasis, while the IL-22 pathway “protects lung function by increasing transepithelial resistance and epithelial cell regeneration and repair” [43]. IL-6 and IL-8 have a more general role in mediating inflammation. E-cig vapor exposure “increases the levels of damaged mtDNA in circulating blood and induces expression of TLR9, which elevates the expression of proinflammatory cytokines in monocytes and macrophages”, consequently leading to atherosclerosis in mice [44]. Similarly, toll-like receptor 9 (TLR-9) activation by circulating mitochondrial DNA (mtDNA) “triggers an intracellular signaling cascade” that has been found to “contribute to elevated arterial pressure and vascular dysfunction in spontaneously hypertensive rats” [44].

The simultaneous downregulation of IL-22 and increased proinflammatory cytokine expression following e-cig exposure demonstrates systemic inflammatory response dysregulation. These decreased levels of immunomodulatory proteins and increased cytokine levels mediate dysregulation of the fundamental host response to inflammatory stimuli. This imbalance of immune system responses due to e-cig exposure suggests greater susceptibility to infection and disease for e-cig users, along with an increased risk of atherosclerosis and epithelial cell degradation [10,43,44]. E-cig exposure disrupts intracellular signaling cascades by dysregulating the production of anti-inflammatory chemokines and cytokines and contributing to vascular dysfunction.

Other studies have investigated the release of endothelin-1 (ET-1), a potent pro-inflammatory cytokine, in response to e-cig exposure. Researchers have isolated endothelial cell-derived microvesicles (EMVs) from e-cig users and determined their effects on NO and ET-1 production within human cerebral microvascular endothelial cells (hCMECs) [45]. ET-1 inhibits eNOS activity and induces vascular smooth muscle inflammation [45]. Therefore, it is expected that e-cig exposure will increase levels of ET-1 as a pro-inflammatory mediator and subsequently decrease eNOS activity (which will further decrease endothelial NO) based on the results presented earlier in this review.

The EMVs isolated from e-cig users “reduced brain microvascular endothelial NO production, decreased eNOS activation [and] enhanced ET-1 production” [45]. These modulatory responses mirror those from previous research studying the effects of CS on EMVs [45]. This research highlights explicitly the damage that occurs to brain microvasculature because of e-cig exposure. However, these findings stress the importance of understanding the mechanisms involved in changes associated with EMV phenotypic composition.

### 7.2. NADPH Oxidase

Researchers have also investigated the role that NADPH oxidase (NOX-2) plays in mediating vascular damage following e-cig exposure with and without the presence of nicotine [46]. Typically, NOX-2 is an enzyme that produces ROS within phagosomes as an immune system defense mechanism; once the phagosomes burst, microbial pathogens are eliminated in respiratory pathways [47]. In this study, they determined that “NOX-2 is responsible for e-cig vaping-induced vascular damage” since mice lacking the NOX-2 gene had preserved endothelial function following nicotine-free vapor exposure, while “wild-type mice exhibited profound endothelial dysfunction” [46].

In further support of these earlier findings, pharmacological inhibition of NOX-2 resulted in lesser ROS formation in the brain’s frontal cortex. Importantly, this study demonstrates that oxidative and inflammatory stress in the brain following e-cigarette exposure is a function of NOX-2 activity. Evidence suggests that the NOX-2-dependent mechanism of oxidative stress may be because once NOX-2 generates ROS, eNOS uncoupling occurs, resulting in mitochondrial dysfunction [48]. The relatively simultaneous increase in ROS and uncoupling of eNOS contribute to the overall increase in oxidative stress.

Even without nicotine, oxidative stress, lipid peroxidation, and dysregulation of endothelial function occur following e-cig exposure. This research supports the notion that even e-cigs without nicotine still induce cerebral and cardiovascular damage to immunological pathways [9]. Furthermore, consistent with previous research, acrolein, a toxic aldehyde, is greatly responsible for the damaging vascular consequences of e-cig usage [14,22,46]. This damage supports the opinion that the aerosolization of e-cigs presents a greater danger than the e-liquid itself because the aerosolization of the e-liquid introduces toxic chemicals that are not present before vaporization [3]. Additionally, an underlying mechanism may occur in which increased oxidative stress occurs because e-cig exposure holistically causes endothelial dysfunction and arterial stiffness at a systemic level [46].

E-cig exposure, regardless of the presence of nicotine, causes a milieu of damage through the increased generation of ROS and increased pro-inflammatory cytokine and chemokine levels, along with holistically dysregulating significant pathways responsible for mediating oxidative stress. E-cig exposure also damages DNA through the downregulation of DNA repair proteins, as increased nicotine metabolites induce methylation and histone modifications [16]. Therapeutic interventions should target e-cig-induced dysregulation by focusing on these major anti-oxidative pathways to promote a cascade of repair throughout systemic vascular pathways [45]. However, more research is still necessary to understand how anti-oxidative and anti-inflammatory molecules can adequately lessen the damages caused by e-cigs.

## 8. Introduction to Bixin

**Figure 3:**
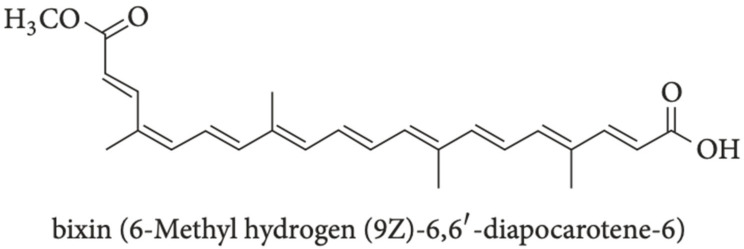
Molecular structure of bixin [50].

Recent research has revealed the promise of using bixin, a natural apocarotenoid component found in *Bixa orellana* seeds within achiote trees in Central and South America [49]. Bixin, a highly conjugated molecule and polyphenol compound as depicted in Figure 3 is an orange-red colored component within *B. orellana* seeds that has been approved by the FDA to be used as an additive and colorant in the food industry in products such as “butter, cheese, soft drinks, and confectionery” [41,50]. Bixin has been found to possess antioxidant, anti-inflammatory, and anticancer characteristics through its actions on the NRF2/HO-1, phosphatidylinositol 3-kinase protein kinase B (PI3K/Akt), and toll-like receptor 4 (TLR4)/NF-κB pathways [51,52,53]. The NRF2 and NF-κB pathways have already been discussed in this review. The actions of bixin on these pathways, along with the PI3K/Akt pathway, introduce bixin as a potential therapeutic agent for the treatment of e-cig-induced damage [52]. The actions of bixin on these pathways will be discussed to showcase research that demonstrates the antioxidative, anti-inflammatory, and anti-cancer actions of bixin.

The NRF2 pathway has been demonstrated as an important cellular defense mechanism against oxidative stress. Bixin-induced antioxidative and anti-inflammatory effects occur in an NRF2-dependent manner by displaying molecular activities including “sacrificial antioxidant [and] excited state quencher [54]. These actions are due to the molecular structure of bixin, where bixin is characterized as an “unsaturated liner carotenoic acid, a dicarboxylic monoester, and a methyl ester with nine conjugated double bonds” with a triglyceride layer [41,55]. These structural components allow bixin to serve as a free radical scavenger through canonical and non-canonical NRF2 induction: its canonical mechanism enhances the stability of NRF2, while the non-canonical mechanism leads to increased NRF2 release, which upregulates HO-1 and “directly quenches ROS in the tissue to attenuate oxidative stress and inflammatory damages” [41].

Bixin inhibits the ubiquitination of NRF2, subsequently enhancing its stability, allowing NRF2 to dislocate from KEAP1, enter the nucleus, and bind to target antioxidant genes [41]. Bixin also stimulates the “interaction between p62 and KEAP1, where p62 serves as an autophagy adaptor protein [31,41]. This interaction increases NRF2 release from KEAP1 because p62 may be a competitive inhibitor for NRF2 binding on KEAP1 [31,41]. In this manner, both mechanisms increase ARE gene expression, subsequently decreasing the presence of ROS in bixin-treated cells [41].

Additionally, the high conjugation of bixin allows for “sweeping free radicals during their transition states” while generating microbial cell membranes’ inactivation [50]. Due to the polyphenol compounds present in annatto seed extract along with the structure of bixin, this substance can “denature the proteins of microbial cell membranes and thus generate their inactivation or death without being affected by the medium pH” [50]. These characteristics of bixin have increased interest in utilizing its properties to treat various conditions and diseases.

Research has shown that bixin suppresses oxidative stress by causing NRF2 translocation in mice with renal damage; this NRF2 translocation allowed for an increase in HO-1 levels and reduction in cytokines such as TNF-α and IL-1β [56]. Bixin nanoparticles decrease levels of TNF-α in a dose-dependent manner [32]. Additionally, bixin inhibited the activation of NF-κB and TLR-4 [56]. Bixin induces antioxidant activity in a dose-dependent manner, where decreased NRF2 and HO-1 levels in mice with high-fat diets were reversed following bixin treatment [51]. Since increased NF-κB activity subsequently represses eNOS expression, enhances iNOS expression, and further overproduces NO, the inhibition of this pathway by bixin will help regulate both the iNOS and eNOS pathways following exposure to e-cig vapor [37]. Bixin increases the stability of the NRF2 pathway and subsequently increases NRF2 production to elicit free radical scavenging and antioxidative activities while inhibiting the activation of pro-inflammatory pathways such as NF-κB [54,56].

Bixin has also been shown to reverse glucocorticoid resistance and suppress allergic airway inflammation by antagonizing the PI3k/Akt pathway within asthmatic mice [57]. The PI3K/Akt pathway is generally responsible for “cell differentiation and proliferation, inflammation, metabolism, and apoptosis” by providing downstream signaling for the tumor growth factor-beta (TGF-β) pathway [57].

Carotenoids have shown reductions in “tumor cell initiation, progression, and metastasis” by blocking this PI3K/Akt pathway through NRF2 overexpression of antioxidant enzymes [52]. This inactivation of the PI3K/Akt pathway will not only inhibit the metastasis of pro-oncogenic activities due to e-cig exposure. For example, EGFR activation has been shown to cause increased cell migration and malignant cell growth through the EGFR/PI3K/mTOR pathway following e-cig exposure [30]. The inhibitory actions of bixin on the PI3K pathway result in the downregulation of P13K targets and a subsequent decrease in metastasis [52]. The effects of bixin on the NRF2, NF-κB, and PI3K allow for decreased metastasis of cancer cell lines [52,54,56]. In this manner, bixin also impacts the cell cycle of tumor cells.

Researchers found that bixin treatment caused cell cycle arrest in Hep3B hepatocellular carcinoma cells, suggesting that bixin induces both extrinsic and intrinsic apoptosis mechanisms [58]. Similarly, another study found that bixin inhibits TNF expression and provides anti-viral properties through biological activity [59]. Additionally, bixin showcases anti-proliferative activity on A549 (lung cancer), HeLa (cervical cancer), and MCF-7 (breast cancer) cell lines [60]. The molecular mechanisms by which bixin exhibits anti-cancer activities remain unclear. Still, bixin induces multi-apoptotic signaling and cytokine downregulation in cancer cells, suggesting the utilization of bixin as a promising therapeutic target for reducing oxidative stress and cancer treatments [60].

## 9. Bixin Nanoparticles

Bixin is an apocarotenoid generally insoluble in water [41]. It showcases high lipophilicity and low bioaccessibility and bioavailability due to its instability in the presence of oxygen, light, heat, and high pHs [41]. Although bixin is a promising therapeutic agent for treating various inflammatory vascular conditions, its physiological properties are not ideal. Multiple kinds of carotenoids have been encapsulated into different forms of nanoparticles, including nanoliposomes, quantum dots, and polymeric nanoparticles, to “enhance the water solubility, storage stability, controlled and sustained release, bioaccessibility, bioavailability, and bioactivity” of these molecules [52].

Bixin has been encapsulated in various nanocarriers to counteract these bioavailability issues [32]. By encapsulating bixin into a water-soluble and biodegradable compound, interactions between bixin and the encapsulating material allow for bixin stabilization and its subsequent release in the desired water-soluble medium [61]. Bixin encapsulation enables it to serve as a common food additive, providing red hues to foods and cosmetic products [41]. The encapsulation of bixin into polymeric nanoparticles has been found to increase bixin stabilization by promoting water solubilization in hydrophilic regions [32].

Nanocarriers have been introduced in the past few decades as a promising therapeutic delivery system for lung issues such as asthma and other forms of pulmonary inflammation [62]. Nanomedicines allow “targeted drug delivery to enhance the efficacy and alleviate adverse side effects” of the administered drug [63]. Nanoparticles also allow for a combinatorial therapeutic approach because they can be loaded with multiple medications or functionalized to provide a more targeted effect for the tissues in question [62]. Traditional drug delivery mechanisms can include intravenous, oral, or inhalation administration.

Modifying these drugs to involve nanoparticle carriers can allow for targeted drug delivery and the reduction in side effects [62]. Nano-drugs can include the nano-modification of currently used drugs or the development of brand-new nano-drugs [62]. Both kinds of nanoparticles have been used to treat asthma [64]. For example, a telomere dendrimer was developed to deliver hydrophobic drugs into the lungs directly, which allowed for a reduction in “allergic pulmonary inflammation” and a decrease in “eosinophils and inflammatory cytokines” [64]. The same approach can be used to develop nanocarriers for the targeted delivery of bixin to treat e-cig-induced damage in lung tissues.

A viable approach for treating e-cig-induced damage would be the delivery of NRF2 activators or NF-κB inhibitors to alleviate oxidative stress [34]. Research has demonstrated the positive effects of nanocarriers on the lungs by designing an “advanced inhalable therapeutic micro/nanoparticle powder delivery method to the lungs via the NRF2/KEAP1 pathway” [34]. This inhalable micro/nanoparticle powder showcased high permeability into lower lung tissues, allowing for the capability to treat inflammation such as acute lung injury and chronic obstructive pulmonary disease in lower areas of the lungs [34]. The same approach could be implemented to treat e-cig-induced inflammation due to the dysregulation of the NRF2 pathway caused by e-cigs. Carotenoid-based nanoparticles have been developed, but there is little research involving the application of bixin nanoparticles specifically [52].

However, bixin-polymeric nanoparticles have been created to treat acute lung injury caused by exposure to cigarette smoke and investigate how bixin may ameliorate ROS generation and increased cytokine levels [32]. These bixin-polymeric nanoparticles possessed antioxidant and anti-inflammatory properties that prevented the typical cigarette smoke-induced increases in ROS levels and leukocyte numbers in bronchoalveolar lavage fluid (BALF) [32]. The resulting amelioration of cigarette-induced damage highlighted the therapeutic potential of utilizing bixin-polymeric nanoparticles to ameliorate the structural and functional damages upon cerebrovascular, cardiovascular, and pulmonary tissues following e-cig exposure.

It is unlikely that bixin nanoparticles can be included in e-cig devices to treat nicotinic e-cig-induced inflammation and damage proactively. While bixin alone is likely to dissolve in the e-liquid chamber found in e-cigs, bixin is unstable at high temperatures; the heating of the coil upon inhalation of the e-cig mouthpiece would degrade bixin and thus eliminate its positive antioxidant effects. Additionally, while bixin nanoparticles have been synthesized to overcome bixin’s lipophilicity and instability at high temperatures, bixin nanoparticles better serve as a treatment for oxidative stress induced by e-cigs rather than as an ingredient in e-cig liquid chambers. E-cigs cause dysregulation of the NRF2, NF-κB, and PI3K pathways, whereas bixin can reactivate/repair these pathways. Furthermore, concurrent delivery of bixin nanoparticles and e-cig aerosol is unlikely to mediate the oxidative stress and dysregulation induced by e-cig usage.

More research must be conducted to properly understand the underlying mechanisms by which bixin exhibits its therapeutic action. Gaining a better understanding will allow for targeted therapeutic approaches to provide systemic relief for those exposed to e-cig vapor. Ideally, these nanoparticles would successfully ameliorate damages induced by e-cig vapor and be used to treat other vascular diseases resulting from ROS generation and oxidative damage.

## 10. Conclusions

The introduction and recent research on e-cigs have resulted in an increased sense of urgency to uncover the underlying mechanisms of e-cig-induced damage. This damage causes several systemic issues, most notably cerebrovascular, cardiovascular, and pulmonary damage, because of increased oxidative stress and DNA damage [45]. E-cig-mediated injuries are associated with the dysregulation of critical anti-inflammatory and anti-oxidative pathways, including the NRF2 pathway and NF-κB pathway, which inherently affect the release of NO and generation of ROS [6,14,46]. Multiple research investigations have concluded that e-cigs are, in fact, not a healthy alternative to conventional cigarettes due to their harmful inflammatory, pro-oncogenic, and deregulatory effects on the body [10,22,48].

The long-term effects of e-cigs are of utmost concern, considering these devices have only been on the market for the past two decades. Adolescents and young adults who have used e-cigs are especially concerned because of the potential for e-cigs to disrupt normal neurological development [3]. More research is necessary to understand the mechanisms by which e-cigarettes can disturb the development of adolescents and induce structural and functional damage to tissues and cells [45].

Recent research has highlighted the potential for utilizing bixin as a promising therapeutic agent for the treatment of e-cig-induced damage [32,41,49,58]. Bixin has showcased anti-oxidative, anti-inflammatory, and anti-cancer activities through relatively unknown mechanisms on the NRF2, NF-κB, and PI3K/Akt pathways [54,56,58]. A greater understanding of the beneficial actions of bixin will allow for efficient treatment for e-cig-induced damage. Bixin nanoparticles will counteract bixin’s innate low bioavailability and promote its advantageous anti-oxidative, anti-inflammatory, and anti-cancer characteristics [32]. Future research should focus on the mechanisms in which bixin exhibits these immunomodulatory effects while exploring the potential of bixin nanoparticles for treating e-cig-induced damage.

## References

[1] Prasad K.N., Bondy S.C. (2022). Electronic cigarette aerosol increases the risk of organ dysfunction by enhancing oxidative stress and inflammation. Drug Chem. Toxicol..

[2] St. Helen G., Havel C., Dempsey D.A., Jacob P., Benowitz N.L. (2016). Nicotine delivery, retention and pharmacokinetics from various electronic cigarettes. Addiction.

[3] Auschwitz E., Almeda J., Andl C.D. (2023). Mechanisms of E-Cigarette Vape-Induced Epithelial Cell Damage. Cells.

[4] Han Y.R., Lee P.I., Pang K.S. (2018). Finding T_max_ and C_max_ in Multicompartmental Models. Drug Metab. Dispos..

[5] Olmedo P., Goessler W., Tanda S., Grau-Perez M., Jarmul S., Aherrera A., Chen R., Hilpert M., Cohen J.E., Navas-Acien A. (2018). Metal Concentrations in e-Cigarette Liquid and Aerosol Samples: The Contribution of Metallic Coils. Environ. Health Perspect..

[6] Lee H.-W., Park S.-H., Weng M.-W., Wang H.-T., Huang W.C., Lepor H., Wu X.-R., Chen L.-C., Tang M.-S. (2018). E-cigarette smoke damages DNA and reduces repair activity in mouse lung, heart, and bladder as well as in human lung and bladder cells. Proc. Natl. Acad. Sci. USA.

[7] Tang M.-S., Wu X.-R., Lee H.-W., Xia Y., Deng F.-M., Moreira A.L., Chen L.-C., Huang W.C., Lepor H. (2019). Electronic-cigarette smoke induces lung adenocarcinoma and bladder urothelial hyperplasia in mice. Proc. Natl. Acad. Sci. USA.

[8] Stanfill S.B., Hecht S.S., Joerger A.C., González P.J., Maia L.B., Rivas M.G., Moura J.J.G., Gupta A.K., Le Brun N.E., Crack J.C. (2023). From cultivation to cancer: Formation of *N* -nitrosamines and other carcinogens in smokeless tobacco and their mutagenic implications. Crit. Rev. Toxicol..

[9] Roxlau E.T., Pak O., Hadzic S., Garcia-Castro C.F., Gredic M., Wu C.-Y., Schäffer J., Selvakumar B., Pichl A., Spiegelberg D. (2023). Nicotine promotes e-cigarette vapour-induced lung inflammation and structural alterations. Eur. Respir. J..

[10] Sayed I.M., Masso-Silva J.A., Mittal A., Patel A., Lin E., Moshensky A., Shin J., Bojanowski C.M., Das S., Akuthota P. (2021). Inflammatory phenotype modulation in the respiratory tract and systemic circulation of e-cigarette users: A pilot study. Am. J. Physiol. Cell. Mol. Physiol..

[11] Yu V., Rahimy M., Korrapati A., Xuan Y., Zou A.E., Krishnan A.R., Tsui T., Aguilera J.A., Advani S., Alexander L.E.C. (2016). Electronic cigarettes induce DNA strand breaks and cell death independently of nicotine in cell lines. Oral Oncol..

[12] Pitzer C.R., Aboaziza E.A., O’reilly J.M., Mandler W.K., Olfert I.M. (2023). Nicotine and Microvascular Responses in Skeletal Muscle from Acute Exposure to Cigarettes and Vaping. Int. J. Mol. Sci..

[13] Litt M.D., Duffy V., Oncken C. (2016). Cigarette smoking and electronic cigarette vaping patterns as a function of e-cigarette flavourings. Tob. Control.

[14] Morris A.M., Leonard S.S., Fowles J.R., Boots T.E., Mnatsakanova A., Attfield K.R. (2021). Effects of E-Cigarette Flavoring Chemicals on Human Macrophages and Bronchial Epithelial Cells. Int. J. Environ. Res. Public Health.

[15] Meernik C., Baker H.M., Kowitt S.D., Ranney L.M., O Goldstein A. (2019). Impact of non-menthol flavours in e-cigarettes on perceptions and use: An updated systematic review. BMJ Open.

[16] Ashoor A., Nordman J.C., Veltri D., Yang K.-H.S., Al Kury L., Shuba Y., Mahgoub M., Howarth F.C., Sadek B., Shehu A. (2013). Menthol Binding and Inhibition of α7-Nicotinic Acetylcholine Receptors. PLoS ONE.

[17] Marques P., Piqueras L., Sanz M.-J. (2021). An updated overview of e-cigarette impact on human health. Respir. Res..

[18] Samburova V., Bhattarai C., Strickland M., Darrow L., Angermann J., Son Y., Khlystov A. (2018). Aldehydes in Exhaled Breath during E-Cigarette Vaping: Pilot Study Results. Toxics.

[19] Xue L., Zhang H., Zhang J., Li B., Zhang Z., Tao S. (2018). Bixin protects against particle-induced long-term lung injury in an NRF2-dependent manner. Toxicol. Res..

[20] Lee J., Tan A.S., Porter L., Young-Wolff K.C., Carter-Harris L., Salloum R.G. (2021). Association Between Social Media Use and Vaping Among Florida Adolescents, 2019. Prev. Chronic Dis..

[21] Thrul J., Gubner N.R., Tice C.L., Lisha N.E., Ling P.M. (2019). Young adults report increased pleasure from using e-cigarettes and smoking tobacco cigarettes when drinking alcohol. Addict. Behav..

[22] Wetzel T.J., Wyatt T.A. (2020). Dual Substance Use of Electronic Cigarettes and Alcohol. Front. Physiol..

[23] Rosenbaum D.P., Hanson G.S. (1998). Assessing the Effects of School-Based Drug Education: A Six-Year Multilevel Analysis of Project D.A.R.E. J. Res. Crime Delinq..

[24] Matos-Ocasio F., Espinoza V.E., Correa-Alfonzo P., Khan A.M., O’Dell L.E. (2021). Female rats display greater nicotine withdrawal-induced cellular activation of a central portion of the interpeduncular nucleus versus males: A study of Fos immunoreactivity within provisionally assigned interpeduncular subnuclei. Drug Alcohol Depend..

[25] Xu X., Li N., Wen J., Yang P., Lu X., Wang Z., He T., Fan Y., Xu B., Ge F. (2023). Specific Inhibition of Interpeduncular Nucleus GABAergic Neurons Alleviates Anxiety-Like Behaviors in Male Mice after Prolonged Abstinence from Methamphetamine. J. Neurosci..

[26] Koob G.F., Arends M.A., Le Moal M. (2014). Psychostimulants. Drugs, Addiction, and the Brain.

[27] Zhao-Shea R., Liu L., Pang X., Gardner P.D., Tapper A.R. (2013). Activation of GABAergic Neurons in the Interpeduncular Nucleus Triggers Physical Nicotine Withdrawal Symptoms. Curr. Biol..

[28] Rebuli M.E., Rose J.J., Noël A., Croft D.P., Benowitz N.L., Cohen A.H., Goniewicz M.L., Larsen B.T., Leigh N., McGraw M.D. (2023). The E-cigarette or Vaping Product Use–Associated Lung Injury Epidemic: Pathogenesis, Management, and Future Directions: An Official American Thoracic Society Workshop Report. Ann. Am. Thorac. Soc..

[29] Klawinski D., Hanna I., Breslin N.K., Katzenstein H.M., Indelicato D.J. (2021). Vaping the Venom: Oral Cavity Cancer in a Young Adult with Extensive Electronic Cigarette Use. Pediatrics.

[30] Wisniewski D.J., Ma T., Schneider A. (2018). Nicotine induces oral dysplastic keratinocyte migration via fatty acid synthase-dependent epidermal growth factor receptor activation. Exp. Cell Res..

[31] Baird L., Yamamoto M. (2020). The molecular mechanisms regulating the KEAP1-NRF2 pathway. Mol. Cell. Biol..

[32] Figueiredo-Junior A.T., Valença S.S., Finotelli P.V., Anjos F.d.F.d., de Brito-Gitirana L., Takiya C.M., Lanzetti M. (2022). Treatment with Bixin-Loaded Polymeric Nanoparticles Prevents Cigarette Smoke-Induced Acute Lung Inflammation and Oxidative Stress in Mice. Antioxidants.

[33] Dang X., He B., Ning Q., Liu Y., Guo J., Niu G., Chen M. (2020). Alantolactone suppresses inflammation, apoptosis and oxidative stress in cigarette smoke-induced human bronchial epithelial cells through activation of Nrf2/HO-1 and inhibition of the NF-κB pathways. Respir. Res..

[34] Muralidharan P., Hayes D., Black S.M., Mansour H.M. (2016). Microparticulate/nanoparticulate powders of a novel Nrf2 activator and an aerosol performance enhancer for pulmonary delivery targeting the lung Nrf2/Keap-1 pathway. Mol. Syst. Des. Eng..

[35] Dong J., Li D., Kang L., Luo C., Wang J. (2023). Insights into human eNOS, nNOS and iNOS structures and medicinal indications from statistical analyses of their interactions with bound compounds. Biophys. Rep..

[36] Liao K., Lv D.-Y., Yu H.-L., Chen H., Luo S.-X. (2021). iNOS regulates activation of the NLRP3 inflammasome through the sGC/cGMP/PKG/TACE/TNF-α axis in response to cigarette smoke resulting in aortic endothelial pyroptosis and vascular dysfunction. Int. Immunopharmacol..

[37] Tejero J., Shiva S., Gladwin M.T. (2019). Sources of Vascular Nitric Oxide and Reactive Oxygen Species and Their Regulation. Physiol. Rev..

[38] Förstermann U., Sessa W.C. (2012). Nitric oxide synthases: Regulation and function. Eur. Heart J..

[39] Krajka-Kuźniak V., Baer-Dubowska W. (2021). Modulation of Nrf2 and NF-κB Signaling Pathways by Naturally Occurring Compounds in Relation to Cancer Prevention and Therapy. Are Combinations Better Than Single Compounds?. Int. J. Mol. Sci..

[40] Phaniendra A., Jestadi D.B., Periyasamy L. (2015). Free radicals: Properties, sources, targets, and their implication in various diseases. Indian J. Clin. Biochem..

[41] Enayati A., Rezaei A., Falsafi S.R., Rostamabadi H., Malekjani N., Akhavan-Mahdavi S., Kharazmi M.S., Jafari S.M. (2023). Bixin-loaded colloidal nanodelivery systems, techniques and applications. Food Chem..

[42] Mohammadi L., Han D.D., Xu F., Huang A., Derakhshandeh R., Rao P., Whitlatch A., Cheng J., Keith R.J., Hamburg N.M. (2022). Chronic E-Cigarette Use Impairs Endothelial Function on the Physiological and Cellular Levels. Arter. Thromb. Vasc. Biol..

[43] Nguyen H.M.-H., Torres J.A., Agrawal S., Agrawal A. (2020). Nicotine Impairs the Response of Lung Epithelial Cells to IL-22. Mediat. Inflamm..

[44] Li J., Huynh L., Cornwell W.D., Tang M.-S., Simborio H., Huang J., Kosmider B., Rogers T.J., Zhao H., Steinberg M.B. (2021). Electronic Cigarettes Induce Mitochondrial DNA Damage and Trigger TLR9 (Toll-Like Receptor 9)-Mediated Atherosclerosis. Arter. Thromb. Vasc. Biol..

[45] Cardenas H.L., Evanoff N.G., Fandl H.K., Berry A.R., Wegerson K.N., Ostrander E.I., Greiner J.J., Dufresne S.R., Kotlyar M., Dengel D.R. (2023). Endothelial-derived extracellular vesicles associated with electronic cigarette use impair cerebral microvascular cell function. J. Appl. Physiol..

[46] Kuntic M., Oelze M., Steven S., Kröller-Schön S., Stamm P., Kalinovic S., Frenis K., Vujacic-Mirski K., Jimenez M.T.B., Kvandova M. (2020). Short-term e-cigarette vapour exposure causes vascular oxidative stress and dysfunction: Evidence for a close connection to brain damage and a key role of the phagocytic NADPH oxidase (NOX-2). Eur. Heart J..

[47] Noreng S., Ota N., Sun Y., Ho H., Johnson M., Arthur C.P., Schneider K., Lehoux I., Davies C.W., Mortara K. (2022). Structure of the core human NADPH oxidase NOX2. Nat. Commun..

[48] Zhang Y., Murugesan P., Huang K., Cai H. (2020). NADPH oxidases and oxidase crosstalk in cardiovascular diseases: Novel therapeutic targets. Nat. Rev. Cardiol..

[49] Ashraf A., Ijaz M.U., Muzammil S., Nazir M.M., Zafar S., Zihad S.N.K., Uddin S.J., Hasnain S., Nayak A.K. (2023). The role of bixin as antioxidant, anti-inflammatory, anticancer, and skin protecting natural product extracted from *Bixa orellana* L.. Fitoterapia.

[50] Quiroz J.Q., Duran A.M.N., Garcia M.S., Gomez G.L.C., Camargo J.J.R. (2019). Ultrasound-Assisted Extraction of Bioactive Compounds from Annatto Seeds, Evaluation of Their Antimicrobial and Antioxidant Activity, and Identification of Main Compounds by LC/ESI-MS Analysis. Int. J. Food Sci..

[51] Xu Z., Kong X.-Q. (2017). Bixin ameliorates high fat diet-induced cardiac injury in mice through inflammation and oxidative stress suppression. Biomed. Pharmacother..

[52] Saini R.K., Prasad P., Lokesh V., Shang X., Shin J., Keum Y.-S., Lee J.-H. (2022). Carotenoids: Dietary Sources, Extraction, Encapsulation, Bioavailability, and Health Benefits—A Review of Recent Advancements. Antioxidants.

[53] Shadisvaaran S., Chin K.-Y., Mohd-Said S., Leong X.-F. (2023). Therapeutic potential of bixin on inflammation: A mini review. Front. Nutr..

[54] de la Vega M.R., Krajisnik A., Zhang D.D., Wondrak G.T. (2017). Targeting NRF2 for Improved Skin Barrier Function and Photoprotection: Focus on the Achiote-Derived Apocarotenoid Bixin. Nutrients.

[55] Oliveira S.D.S.D.C., Araújo R.D.C., da Silva G.A., Leitão J.H., Sousa S.A.B.d.S., Fonseca L.P., Carvalho J.C.T., Cantuária P., Hage-Melim L.I.d.S., Ferreira I.M. (2022). *Bixa orellana* L. from northern Brazil: Morphological analysis, phenolic content, antioxidant and antibacterial activities. Braz. J. Bot..

[56] Ma J.-Q., Zhang Y.-J., Tian Z.-K., Liu C.-M. (2021). Bixin attenuates carbon tetrachloride induced oxidative stress, inflammation and fibrosis in kidney by regulating the Nrf2/TLR4/MyD88 and PPAR-γ/TGF-β1/Smad3 pathway. Int. Immunopharmacol..

[57] Zhu Y., Sun D., Liu H., Sun L., Jie J., Luo J., Peng L., Song L. (2021). Bixin protects mice against bronchial asthma though modulating PI3K/Akt pathway. Int. Immunopharmacol..

[58] Kumar Y., Phaniendra A., Periyasamy L. (2018). Bixin Triggers Apoptosis of Human Hep3B Hepatocellular Carcinoma Cells: An Insight to Molecular and In Silico Approach. Nutr. Cancer.

[59] Muthusamy K., Ramasamy G., Ravikumar C., Natesan S., Muthurajan R., Uthandi S., Kalyanasundaram K., Tiwari V. (2024). Exploring bixin from *Bixa orellana* L. seeds: Quantification and in silico insights into its anti-cancer potential. J. Biomol. Struct. Dyn..

[60] Kusmita L., Franyoto Y.D., Mutmainah M., Puspitaningrum I., Nurcahyanti A.D.R. (2022). *Bixa orellana* L. carotenoids: Antiproliferative activity on human lung cancer, breast cancer, and cervical cancer cells in vitro. Nat. Prod. Res..

[61] Figueiredo-Junior A.T., Anjos F.d.F.d., Brito F.d.C.d.M., Viana V.G.F., Valença S.S., Lanzetti M., Finotelli P.V. (2021). Bixin loaded on polymeric nanoparticles: Synthesis, characterization, and antioxidant applications in a biological system. Appl. Nanosci..

[62] Parhi P., Mohanty C., Sahoo S.K. (2012). Nanotechnology-based combinational drug delivery: An emerging approach for cancer therapy. Drug Discov. Today.

[63] Liu D., Long M., Gao L., Chen Y., Li F., Shi Y., Gu N. (2022). Nanomedicines Targeting Respiratory Injuries for Pulmonary Disease Management. Adv. Funct. Mater..

[64] Kenyon N.J., Bratt J.M., Lee J., Luo J., Franzi L.M., Zeki A.A., Lam K.S. (2013). Self-assembling nanoparticles containing dexamethasone as a novel therapy in allergic airways inflammation. PLoS ONE.

